# Evolution of Zeolite Crystals in Self-Supporting Faujasite Blocks: Effects of Hydrothermal Conditions

**DOI:** 10.3390/ma12121965

**Published:** 2019-06-18

**Authors:** Liuliu Guan, Zhuangzhuang Wang, Duyou Lu

**Affiliations:** College of Materials Science and Engineering, Nanjing Tech University, Nanjing 211800, China; gllyoyo123@njtech.edu.cn (L.G.); wangzz@njtech.edu.cn (Z.W.)

**Keywords:** metakaolin, geopolymer, hydrothermal treatment, self-supporting zeolite blocks, faujasite

## Abstract

In order to prepare self-supporting faujasite (FAU) zeolite, a self-supporting zeolite block was synthesized in situ by hydrothermal treatment of a metakaolin base geopolymer. The effects of hydrothermal conditions such as hydrothermal alkalinity, temperature and time on the phase composition, microstructure and mechanical strength of the hydrothermal samples were investigated and evidenced by a series of characterization methods such as X-ray diffraction (XRD), scanning electron microscopy (SEM), and Brunauer-Emmet-Teller (BET). The results showed that a self-supporting faujasite block could be obtained by hydrothermal treatment of the geopolymer block in 2 M NaOH solution at 90 °C for 24 h, which had high crystallinity, regular morphology and high compressive strength. The self-supporting zeolite block had a compressive strength of 11.7 MPa, a pore volume of 0.24 cm^3^/g, and an average pore diameter of 7.86 nm. The specific surface area and the microporous specific surface area of the self-supporting faujasite blocks were 80.36 m^2^/g and 19.7 m^2^/g, respectively.

## 1. Introduction

Heavy metal pollution seriously harms the environment and human health [1,2,3]. How to effectively control heavy metal pollution is an urgent problem that needs to be solved. Zeolite has a three-dimensional skeleton and porous structure, and has strong adsorption capacity. It is a widely used adsorbent material for removing such harmful pollutants [4]. Zeolites also have a wide range of applications in catalysis. Catizzone et al. [5] showed that the crystal size strongly influences the catalytic effect during the vapour-phase dehydration of methanol to dimethyl ether on ferrierite (FER) zeolite crystals. Valtchev et al. [6] gave an overview of the characteristics and productive ways of hierarchical zeolites materials. However, in practical applications, granular or powdered zeolites often cause secondary pollution due to difficulty in recovery. Therefore, the preparation of self-supporting bulk zeolite with a certain strength is an effective way to solve such problems [7].

Geopolymer refers to a zeolite-like three-dimensional network gel with amorphous or quasi-crystalline characteristics formed by polymerization of a silicon oxytetrahedron and an aluminoxy tetrahedron under high alkaline conditions [8,9,10]. Under the appropriate hydrothermal conditions, Na_2_O/SiO_2_ molar ratio and H_2_O/Na_2_O molar ratio [11], the geopolymer gel can be crystallized in situ to form zeolite [9]. Cui et al. [12,13] used sol-gel method to synthesize high-purity and high-activity Al_2_O_3_-SiO_2_ powder, and used it as raw material to prepare geopolymer. Then, a self-supporting NaA zeolite membrane with excellent pervaporation performance was prepared from geopolymer by hydrothermal method, and the evolution of zeolite-like structures was investigated. Lee et al. [4,7,14] synthesized self-supporting zeolite blocks containing Na-P1 zeolite and sodalite phase by hydrothermal treatment of fly ash-slag based geopolymer, the strength could reach 16.6 MPa, and confirmed that the type and ratio of starting materials significantly affected the formation and evolution of zeolite crystals in self-supporting zeolite blocks. Liguori et al. [15] obtained a self-supporting zeolitic material with a hierarchical porosity by combining zeolite crystallization with a foaming process initiated by silicon and found that the silicon content, the relative humidity and curing time had great effect on the nucleation and growth of zeolite phases. Chen et al. [16] had successfully synthesized various low-silica nanozeolites including FAU, cancrinite (CAN), Linde-Type A (LTA), and sodalite (SOD) by exploring geopolymeric Na-Al-Si-H_2_O quaternary phase diagram. Compared to NaA(4.1 Å) and Na-P1(3.5 Å) [17,18], faujasite (FAU) has a characteristic pore size of 7.4 Å and a unique supercage structure, which is widely used in the field of heavy metal ion adsorption [19,20]. Sutarno et al. [21] hydrothermally synthesized FAU zeolite with the raw materials of fly-ash at 100 °C alkaline solution by reflux with HCL and fusion with NaOH solution and studied the formation and transformation of FAU by variation of NaOH/fly ash weight ratios and hydrothermal times. 

In this paper, in order to prepare self-supporting FAU zeolite, the self-supporting zeolite block was synthesized in situ by hydrothermal treatment of metakaolin based geopolymer. X-ray diffraction (XRD), scanning electron microscopy (SEM), Brunauer-Emmet-Teller (BET) adsorption and other characterization methods were used to investigate and evidence the impact of different hydrothermal conditions such as hydrothermal alkalinity, hydrothermal temperature and hydrothermal time on the formation and evolution of FAU crystals.

## 2. Experimental

### 2.1 Materials

A raw metakaolin (MK) sample with a volume average particle diameter of 5.09 μm was obtained from Tao Jinfeng Kaolin Co., Ltd. in Taining, Fujian, China. The chemical composition data of metakaolin determined by X-ray Fluorescence (XRF) are given in Table 1. The alkali activator used was a mixed solution of water glass and sodium hydroxide (purity ≥ 96%). The Ms is defined as the molar ratio of SiO_2_ to Na_2_O. An appropriate amount of solid sodium hydroxide and deionized water were added to industrial water glass (Ms = 3.1) and mixed to obtain a modified water glass (solid content = 37%, Ms = 1.0). The modified water glass was allowed to stand for 24 h before use.

### 2.2. Sample Preparation

The metakaolin and modified water glass were prepared according to the FAU theoretical formula Na_2_Al_2_Si_3.3_O_10.6_·7H_2_O (JCPDS card No. 12-0228) in a ratio of n(SiO_2_)/n(Al_2_O_3_) = 3.4. The metakaolin and the modified water glass were mixed with a slurry mixer, stirred slowly for 5 minutes, and stirred for 5 min. Then, the stirred slurry was injected into the mold (20 × 20 × 20 mm^3^) and the mold was coated with plastic wrap. The mold was then removed after curing for 24 h in a standard curing box of (20 ± 2) °C, relative humidity RH > 90%. 

The demolded test piece was placed in a hydrothermal kettle and hydrothermally treated. The effects of process parameters such as hydrothermal alkalinity, hydrothermal temperature and hydrothermal time of hydrothermal solution (NaOH solution) on the formation and evolution of zeolite crystals were studied. The samples and the related hydrothermal parameters are listed in Table 2.

The hydrothermally treated test block was thoroughly washed in deionized water until a pH value of the wash water was below 10. The cleaned test blocks were then dried at 110 °C for 10 h and the degree of crystallization, morphology and specific surface area of the blocks obtained after being dried were measured. The undried sample after cleaning was further cured at room temperature for 3 days and 28 days for compressive strength measurement. Table 2 Samples and tested hydrothermal conditions. M in the whole manuscript is defined as mol/L

### 2.3. Materials Characterization

The compressive strength of the sample was tested using a universal testing machine (WYH-200, Hualong Testing Instrument Co., Ltd., Shanghai, China). The powder sample was collected on one of the sides of the cubes and was analyzed by X-ray diffractometer (XRD) (Smartlab-3 kw, Rigaku, Japan) (Cu-Kα, tube voltage 40 kV, tube current 30 mA), step size 0.02°, scanning range 5~50°, scanning speed 10°/min. The newly fractured sample was coated with Au, and the shape of the sample was analyzed by SU8200 ultra-high-resolution thermal field emission scanning electron microscope (SEM) (Hitachi High-Technologies Corporation, Tokyo, Japan). The acceleration voltage was 15 kv. Nitrogen adsorption tests were performed using an nitrogen adsorber (ASAP 2020, Micromeritics, Norcross, Georgia, GA, USA) and tested at a relative pressure (P/P0) from 0 to 0.99. The specific surface area (SSA) of the sample was calculated from the adsorption data of N_2_ using the standard Brunauer-Emmet-Teller (BET) method.

## 3. Results and Discussion

### 3.1. Effect of Alkalinity 

**Figure 1:**
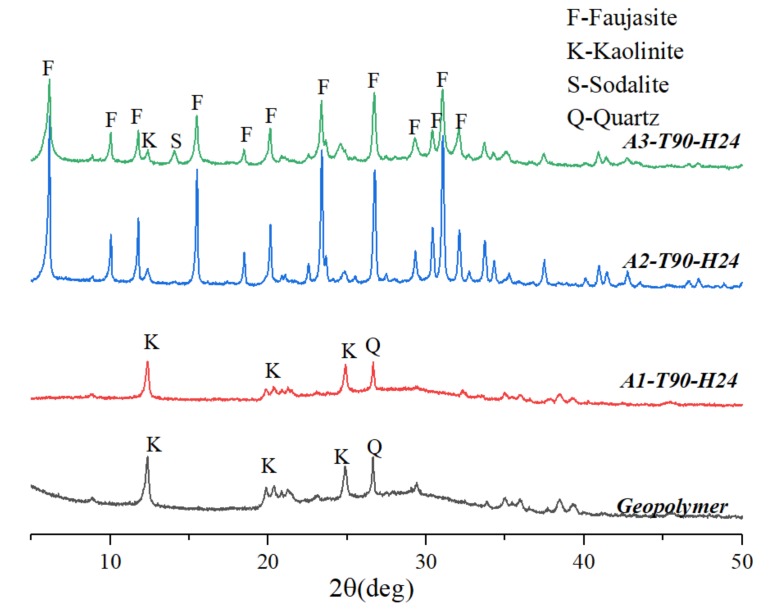
XRD patterns of samples synthesized at different hydrothermal alkalinity.

Figure 1 is an XRD pattern of a hydrothermal sample for 24 h in a different alkalinity NaOH solution at a temperature of 90 °C. When the solution had a basicity of 1 M, the diffraction spectrum of the sample (A1-T90-H24) was similar to that of the geopolymer without hydrothermal treatment. A1-T90-H24 was mainly the dispersion peak of N-A-S (H) gel, but the background of the diffraction spectrum was obviously reduced. It was shown that when the hydrothermal alkalinity was low, it was insufficient to form an XRD-identifiable zeolite crystal. When the solution had a basicity of 2 M, the degree of crystallization of the sample was remarkably increased, and a large number of faujasite crystals were formed. However, when the alkalinity of the hydrothermal solution continued to increase to 3 M, the diffraction intensity of the faujasite in the sample (A3-T90-H24) was significantly lowered, and the sodalite phase appeared. It indicated that the alkalinity of the hydrothermal solution was too high to obtain the faujasite crystal.

**Figure 2:**
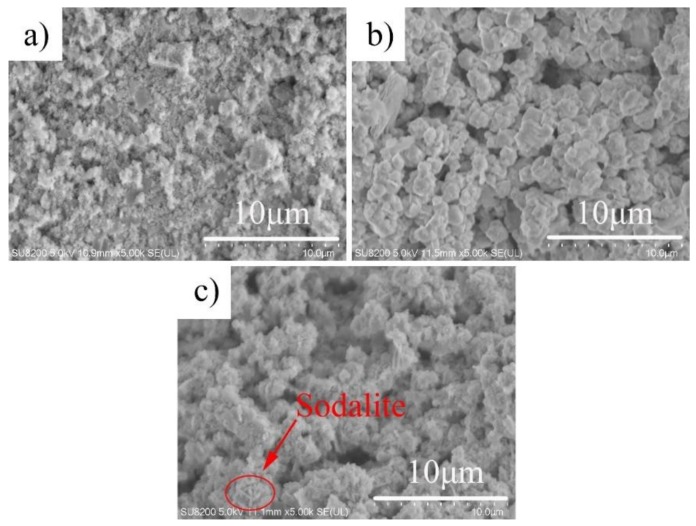
SEM micrographs of the samples A1-T90-H24 (**a**); A2-T90-H24 (**b**) and A3-T90-H24 (**c**).

The SEM image of the hydrothermal sample under different alkalinity of hydrothermal solution is shown in Figure 2. As shown in Figure 2a, when the solution had a basicity of 1 M, the sample (A1-T90-H24) mainly consisted of amorphous gel, which was consistent with its XRD diffraction spectrum. When the alkalinity of the hydrothermal solution was low, the geopolymer gel could not depolymerized. In this state, the cations was hard to enter the crystal lattice, and the new structure could not form due to the absence of crystallization [18]. When the alkalinity of the hydrothermal solution was 2 M, an octahedral faujasite crystal was formed in the sample (A2-T90-H24), and the morphology was regular. When the concentration of NaOH solution increased to 3 M, the faujasite agglomerates in a spherical shape. The crystal particles became smaller, and the formation of hairy spherical sodalite crystals were observed, similar to other studies [7]. The results showed that too high a hydrothermal solution alkalinity cause the faujasite crystals to be eroded and lead to the formation of sodalite crystals.

### 3.2. Effect of Hydrothermal Temperature

**Figure 3:**
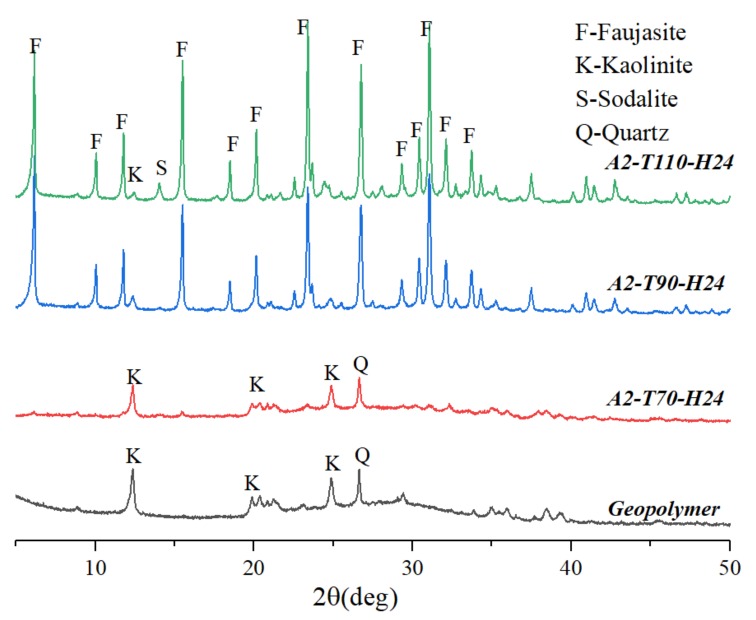
XRD patterns of samples synthesized at different hydrothermal temperature.

Figure 3 is an XRD pattern of a hydrothermal sample for 24 h at different hydrothermal temperatures in a 2 M NaOH solution. The hydrothermal sample (A2-T70-H24) was mainly an amorphous phase at a hydrothermal temperature of 70 °C, which was not much different from the sample (geopolymer) without hydrothermal treatment. It indicated that the geopolymer gel could not be converted into zeolite crystals when the hydrothermal temperature was low. As the hydrothermal temperature increased to 90 °C, the amorphous phase in the sample (A2-T90-H24) decreased, and a large amount of faujasite crystals were formed, remarkably improving the degree of crystallization. When the hydrothermal temperature was suitable, it could provide enough energy to promote nucleation, accelerating the crystallization process [18]. When the temperature was 110 °C, the intensity of the faujasite diffraction peaks in the sample (A2-T110-H24) increased, but the sodalite crystal appeared. Above results confirmed that when the hydrothermal temperature was too high, it was not conducive to the preparation of self-supporting faujasite blocks.

**Figure 4:**
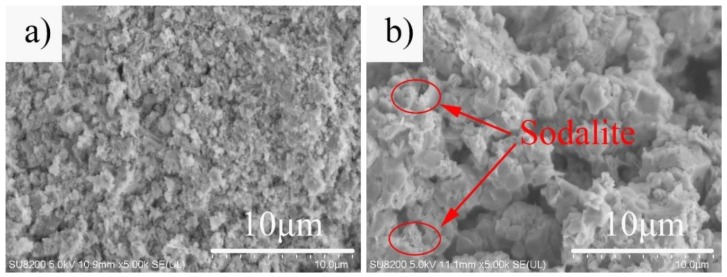
SEM micrographs of the samples A2-T70-H24 (**a**) and A2-T110-H24 (**b**).

The SEM image of the sample at different hydrothermal temperatures is shown in Figure 4. When the hydrothermal temperature was 70 °C, the sample (A2-T70-H24) was composed of an amorphous gel (Figure 4a). When the hydrothermal temperature reached 90 °C, a large amount of faujasite crystals were formed in the sample (A2-T90-H24) (Figure 2b). But when the temperature was further increased to 110 °C, the edge of the zeolite crystals in the sample (A2-T110-H24) became rough and irregular, the crystal grains became small, and hair-like sodalite crystals were formed (Figure 4b).

### 3.3. Effect of Hydrothermal Time

**Figure 5:**
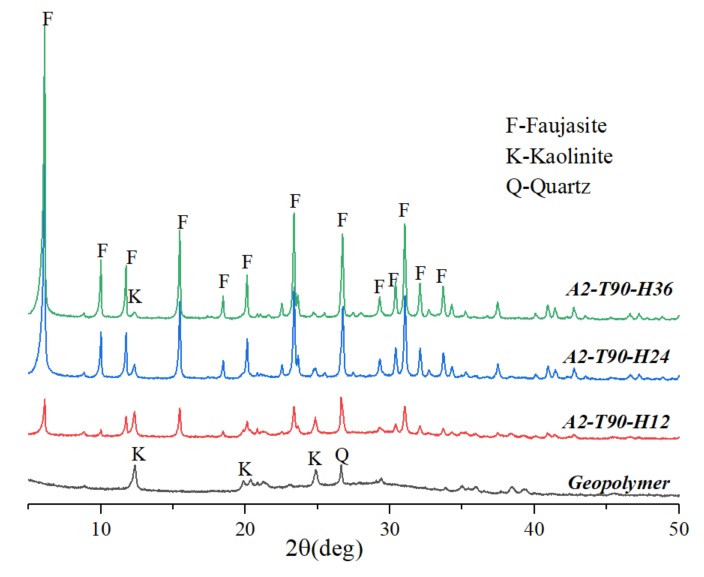
XRD patterns of samples synthesized at different hydrothermal time.

Figure 5 is an XRD pattern of a hydrothermal sample for different hydrothermal time in a 2 M NaOH solution at a temperature of 90 °C. The geopolymer sample without hydrothermal treatment was mainly an amorphous phase. When the hydrothermal time was 12 h, the diffraction peak of the faujasite crystal with relatively weak diffraction intensity appeared in the sample (A2-T90-H12), but there were still other obvious peaks. With a hydrothermal time of 24 h, the amount of formed crystals increased, and the crystallization degree of the sample (A2-T90-H24) was significantly improved. When the hydrothermal time was extended to 36 h, the intensity of the faujasite diffraction peak in the sample (A2-T90-H36) further increased. However, the sodalite crystal phase did not appear in the sample (A2-T90-H36) as in the sample (A3-T90-H24) and the sample (A2-T110-H24).

**Figure 6:**
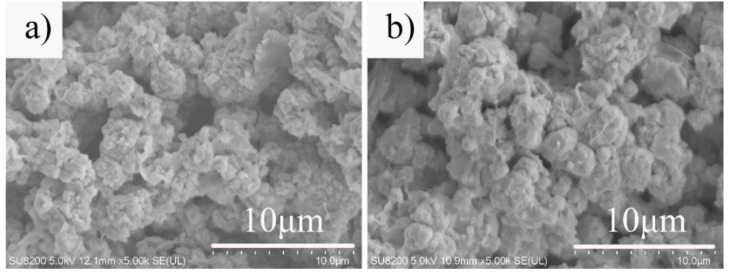
SEM micrographs of the samples A2-T90-H12 (**a**) and A2-T110-H36 (**b**).

The SEM image of the hydrothermal sample at different hydrothermal times is shown in Figure 6. When the hydrothermal time was 12 h, the sample (A2-T90-H12) was mainly a fine-grained faujasite crystal and an amorphous gel (Figure 6a). It showed that the crystallization degree of faujasite was not high as the hydrothermal time was insufficient, which was consistent with the XRD diffraction spectrum. When the hydrothermal time increased to 24 h, a large number of well-formed faujasite crystals (Figure 2b) could be observed in the sample (A2-T90-H24). When the hydrothermal time increased to 36 h, the edges of the faujasite crystals in the sample (A2-T90-H24) became rough (Figure 6b), and the surface showed a significant dissolution phenomenon. Moreover, the crystal particles agglomerated and became bigger. It indicated that a longer hydrothermal treatment was conducive to the formation of faujasite crystals.

### 3.4. Compressive Strength Analysis

**Figure 7:**
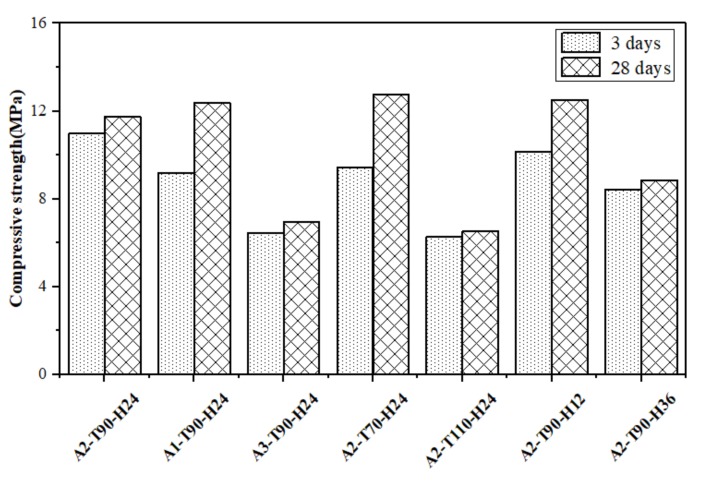
Compressive strength of self-supporting zeolite blocks.

**Figure 8:**
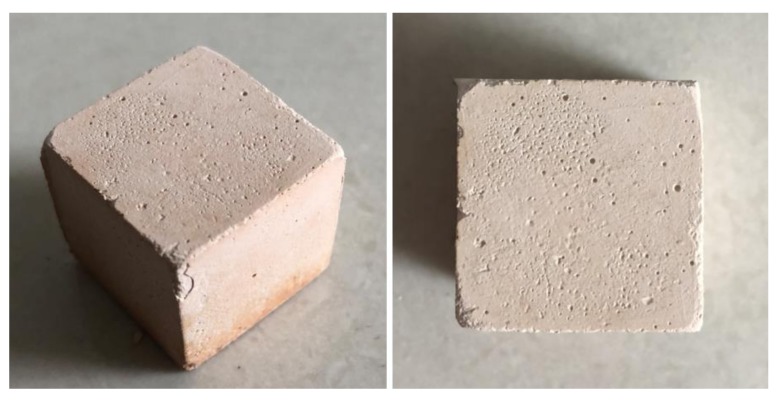
Pictures of sample A2-T90-H24.

The 3 days and 28 days compressive strength of the hydrothermal sample is shown in Figure 7. The 3 days compressive strengths of samples A1-T90-H24 and A2-T70-H24 reached 9.2 MPa and 9.4 MPa, respectively, while the 3d compressive strength of samples A2-T90-H24 with better crystallization degree was 11.0 MPa. The results of compressive strength development were consistent with the previous report [22]. The 28 days compressive strength of the sample A2-T90-H24 was 11.7 MPa, which was lower than that of the samples A1-T90-H24 and A2-T70-H24. It indicated that the compressive strength development of the geopolymer gel was superior than that of the zeolite crystal. The 3 days compressive strength of samples A3-T90-H24, A2-T110-H24 and A2-T90-H36 were lower than those of sample A2-T90-H24, probably due to the formation of sodalite crystals and the dissolution of faujasite crystals. Although longer hydrothermal treatment favored the formation of faujasite crystals, it was not conducive to the increase in compressive strength of the blocks. Therefore, excessive alkalinity, excessive hydrothermal temperature or excessive hydrothermal time would result in a decrease in the compressive strength of the sample. The pictures of sample A2-T90-H24 are shown in Figure 8.

### 3.5. Pore Structure Analysis

**Figure 9:**
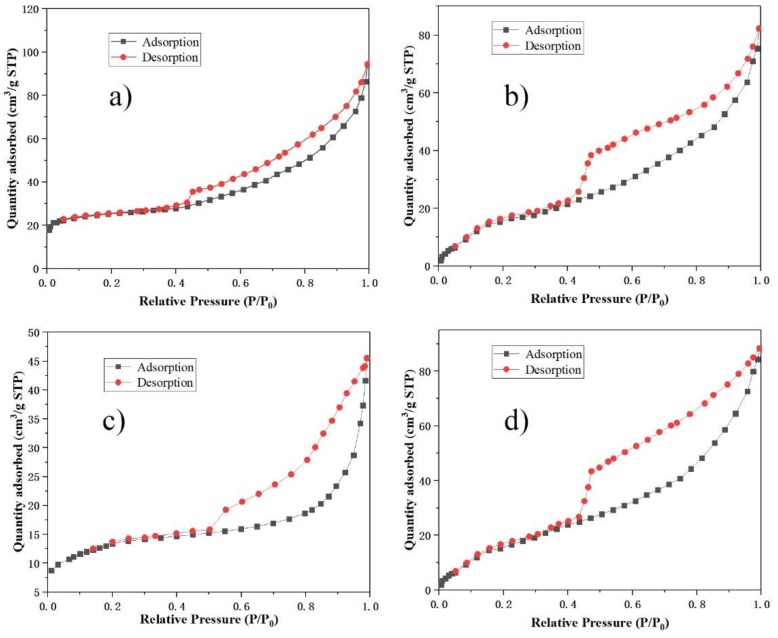
N_2_ adsorption and desorption isotherms of samples A2-T90-H24 (**a**); A3-T90-H24 (**b**); A2-T110-H24 (**c**) and A2-T90-H3 (**d**).

The N_2_ adsorption and desorption isotherms of some samples are illustrated in Figure 9. The isotherms for these samples represent type IV isotherms with an H3-type hysteresis loop in the International Union of Pure and Applied Chemistry (IUPAC) classification, characteristic of mesoporous materials. T-plot method was used for the estimation of micropore area, and Barrett-Joyner-Halenda (BJH) analysis was used for the estimation of average pore diameter. The data estimated by the t-plot method and the BJH method were only for reference, and there was a certain error compared with the actual value. The pore structure parameters of some samples are shown in Table 3. The specific surface area of sample A2-T90-H24 reached 80.36 m^2^/g, which was higher than that reported by other authors [22,23]. Sample A2-T90-H24 had a micropore area of 19.7 m^2^/g, a pore volume of 0.24 cm^3^/g, and an average pore diameter (interparticle space) of 7.86 nm. In comparison with the sample A2-T90-H24, the specific surface area and pore volume of the samples A3-T90-H24, A2-T110-H24 and A2-T90-H36 were significantly lower, and the average pore diameter was bigger. It was shown that too high a hydrothermal alkalinity, temperature or time were not conducive to the development of pore structure parameters of self-supporting faujasite blocks.

## 4. Conclusions

In our study, the effect of different hydrothermal conditions on the crystal evolution of geopolymer hydrothermal conversion of faujasite was investigated. The results showed that the geopolymer block could be hydrothermally treated in 2 M NaOH solution at 90 °C for 24 h to obtain a self-supporting faujasite block with high crystallinity, regular morphology and high compressive strength. The self-supporting zeolite block had a compressive strength of 11.7 MPa, a pore volume of 0.24 cm^3^/g, and an average pore diameter of 7.86 nm. The specific surface area and microporous specific surface area of the self-supporting faujasite blocks were 80.36 m^2^/g and 19.7 m^2^/g, respectively. In the case of hydrothermal treatment of 1 M NaOH hydrothermal solution, hydrothermal temperature of 70 °C or hydrothermal time of 12 h, the amorphous gel phase in the geopolymer could not meet the crystallization requirements of the zeolite. In the case of hydrothermal treatment of 3 M NaOH hydrothermal solution, hydrothermal temperature of 110 °C or hydrothermal time of 36 h, the formed zeolite crystals would be dissolved in the alkaline solution. These situations also promote the formation of sodalite crystals, and result in the reduction of compressive strength and pore structure parameters of the self-supporting blocks. Therefore, suitable hydrothermal conditions are essential in the process of hydrothermal formation of zeolite crystals by geopolymer. By adjusting hydrothermal conditions, self-supporting zeolite blocks with different crystal phase content, compressive strength and pore structure can be prepared and we can thus realize its multi-functional application.

## Figures and Tables

**Table 1:** Chemical compositions of raw materials by XRF analysis (mass, %).

Sample	**SiO_2_**	**Al_2_O_3_**	**CaO**	**Fe_2_O_3_**	**K_2_O**	**TiO_2_**	**MgO**	**SO_3_**	**MnO**	**P_2_O_5_**	**Na_2_O**	**LOI**
MK	48.43	38.68	1.95	0.972	0.503	0.178	0.169	0.156	0.0654	0.0316	0.0316	8.76

**LOI:** loss on ignition.

**Table 2:** Samples and tested hydrothermal conditions.

Sample	Hydrothermal Alkalinity/mol·L^−1^	Hydrothermal Temperature/°C	Hydrothermal Time/h
Geopolymer	0	0	0
A1-T90-H24	1	90	24
A2-T90-H24	2	90	24
A3-T90-H24	3	90	24
A2-T70-H24	2	70	24
A2-T110-H24	2	110	24
A2-T90-H12	2	90	12
A2-T90-H36	2	90	36

**Table 3:** Pore characteristics of self-supporting zeolite blocks.

Sample	BET Surface Area (m^2^/g)	Micropore Area (m^2^/g)	Pore Volume (cm^3^/g)	Average Pore Diameter (nm)
A2-T90-H24	80.36	19.7	0.24	7.86
A3-T90-H24	53.16	11.8	0.19	9.27
A2-T110-H24	44.42	9.6	0.16	11.36
A2-T90-H36	60.42	14.3	0.21	8.94
